# Four clinical and biological phenotypes in antiphospholipid syndrome: a cluster analysis of 174 patients with antinuclear antibody tests

**DOI:** 10.3389/fimmu.2024.1361062

**Published:** 2024-02-19

**Authors:** Marie Ottavi, Pierre Toulon, Barbara Casolla, Nihal Martis

**Affiliations:** ^1^ Internal Medicine Department, University Hospital of Nice, Cote d’Azur University, Nice, France; ^2^ Haematology Department , University Hospital of Nice, Cote d’Azur University, Nice, France; ^3^ Stroke Unit, UR2CA-URRIS Neurology, University Hospital Pasteur 2, Cote d’Azur University, Nice, France; ^4^ Mediterranean Centre for Molecular Medicine, Control of Gene Expression (COdEX), INSERM U1065, Nice, France

**Keywords:** antibodies, antiphospholipid, antiphospholipid syndrome, lupus erythematosus, systemic, heart disease risk factors, connective tissue diseases

## Abstract

**Introduction:**

Antiphospholipid syndrome (APS) is an autoimmune thrombotic disease with various systemic presentations. This study aimed to identify homogeneous groups of patients based on a non-supervised hierarchical cluster analysis and assess the rate of relapse associated with antinuclear antibodies (ANA).

**Methods:**

This retrospective observational study enrolled patients, over a 90-month period, who had APS as defined by the 2006 Sydney classification criteria, and for whom ANA workup was performed. Agglomerative unsupervised hierarchical clustering was conducted to classify patients into subgroups using 24 variables reflecting a range of clinical and biological baseline features associated with APS.

**Results:**

Hundred and seventy-four patients were included and were categorized into four phenotypes. Cluster 1 (n=73) associated mostly middle-aged men with risk factors for cardiovascular disease. Obstetrical APS with low-risk thrombosis made up cluster 2 (n=25). Patients with venous thromboembolism (VTE), microvascular findings and double/triple positive APL antibodies (50%) were represented in cluster 3 (n=33). Whereas cluster 4 (n=43) characterized a predominantly female subpopulation with positive ANA and systemic lupus (n=23) that exhibited a high thrombotic risk and more frequent relapses (n=38) (p<0.001).

**Conclusions:**

This study identified four homogenous groups of patients with APS listed as: i) cardiovascular and arterial risk, ii) obstetrical, iii) VTE and microvascular, and iv) ANA-positive APS. We found that ANA-positivity was associated with higher rates of relapse. Applying ANA status to classification criteria could constitute a novel approach to tailoring management for APS, based on phenotypic patterns and risk assessment.

## Graphical Abstract

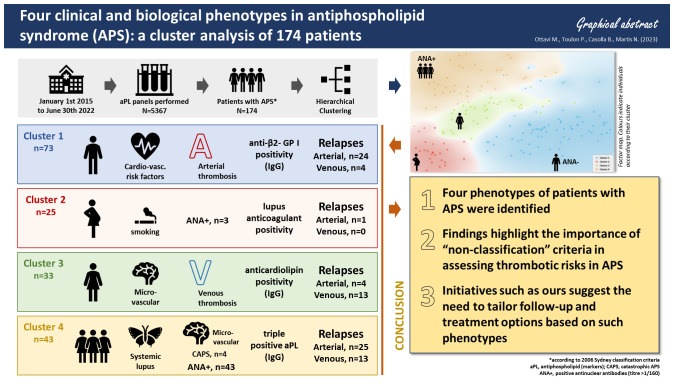

## Introduction

Antiphospholipid syndrome (APS) is an autoimmune disease with systemic features associated with arterial, venous, or microvascular thrombosis, pregnancy morbidity in patients with persistent antiphospholipid antibodies (aPL) (1). The latter refer to: lupus anticoagulant (LA), anticardiolipin (aCL) and anti-β2-glycoprotein I (aβ2-GP.I). Until recently, classification of APS relied on the Sapporo criteria (2) that were revised in 2006 (also known as the Sydney criteria) (1). Such criteria lacked specificity and did not account for clinical phenotypes outside of the obstetrical context (previously referred to as “non-criteria” features). Recently, the *American College of Rheumatology* (ACR) and the *European League Against Rheumatism* (EULAR) published an updated approach for the classification of APS so as to provide “high specificity and a strong foundation for future research” (3). In reality, patients present with a wide range of manifestations with different risk profiles that do not necessarily reflect established clinical and/or biological domains. This may lead to dilemmas in decision-making, when classification criteria are used as diagnostic aids (4).

Previous studies have attempted to identify subgroups of patients with APS with common features and prognoses (5–12). Moreover, unsupervised statistical methods have been used to determine phenotypes in populations of individuals with APS (6–11). Hierarchical cluster analysis is one of such processes that can be used to assess the pertinence of clinical domains (from the newly established classification criteria) as well as relevant clinical and biological features commonly associated with APS. Among such features is the analysis of antinuclear antibodies (ANA), indiscriminately associated or not with well-defined systemic autoimmune diseases. The clinical value of ANA positivity therefore needs to be examined since it has not only been found to be associated with higher morbidity in the obstetrical context but also with a higher rate of thrombotic relapses in comparison to ANA-negative APS patients (13–15). ANA, however, do not appear in classification criteria domains (1–3).

We hypothesize that ANA-positivity is associated with a specific phenotype of APS. The aim of this study was to identify homogeneous groups of APS patients based on a hierarchical cluster analysis. We furthermore chose to assess differences in relapse-rates accordingly in patients with a complete ANA workup.

## Materials and methods

### Study population and definitions

This was a retrospective observational study that included patients treated at the University Hospital of Nice, France, over a period spanning from January 1^st^ 2015 au June 30^th^ 2022. Subjects aged 17 years and above were identified via a database search for antiphospholipid positivity, and those meeting the 2006 Sydney classification criteria for APS were selected for the study (2) (**Supplementary Data**, **Supplementary Table S2**). Patients with duplicate and/or missing data – as well as those that lacked an immunological work-up with ANA – were excluded from the study even if APS criteria were met.

Catastrophic antiphospholipid syndrome (CAPS) was defined according to Asherson et al. (16). Whereas, “microvascular involvement” referred to livedo racemosa, livedoid vasculopathy, aPL nephropathy and/or pulmonary hemorrhage based on the latest classification criteria (3). Diagnoses of systemic lupus erythematosus (SLE) were those made in the clinical setting and were retrospectively required to meet any of the classification criteria for the disease (17). ANA were considered positive for titres above 1/160 (expressed as “≥1/160” in the manuscript). Immunological markers with their ranges are presented in the **Supplementary Data** section (**Supplementary Table S3**).

Data were collected from patients’ digital medical files and included findings such as demographics, clinical characteristics (including comorbidities, initial symptoms, microvascular involvement and cardio- and cerebro-vascular risk factors) and laboratory workup. Focus was given to hematological and immunological features such as: LA, aCL and aβ2-GP.I with their IgM and IgG antibody isotypes, platelet counts, creatinine and kidney function, ANA and complement function tests. The estimated glomerular filtration rate (eGFR) was based on the *Modification of Diet in Renal Disease Study Group (MDRD)* method (18). Immuno-assays with their associated cut-off values are provided as **Supplementary Data** (**Supplementary Table S3**).

“Double-positive aPL” was defined as the positivity of any combination of two aPL biomarkers irrespective of immunoglobulin isotype for aCL and/or aβ2-GP.I, whereas “triple positivity” referred to the presence of all three aPL biomarkers (irrespective of immunoglobulin isotype).

Based on previous studies (6–11) (**Supplementary Data**, **Supplementary Table S4**), clinical relevance and the recent 2023 ACR/EULAR classification criteria for APS (3), we chose the following 24 categories for the hierarchical cluster analysis: age of >50 years, gender, arterial hypertension, *diabetes mellitus*, dyslipidemia, smoking, obesity, SLE, initial arterial and/or venous thrombotic event, cardiac valve involvement, microvascular involvement, CAPS, obstetric features, LA, IgM and IgG aCL, IgM and IgG aβ2-GP.I, thrombocytopenia <130*10^9^/L, eGFR <60 mL/mn/1.73m², positive ANA, anti-SSA and anti-dsDNA antibody positivity.

### Statistical analysis

Agglomerative unsupervised hierarchical clustering based on the Ward method (for linkage) was performed using the 24 previously stated variables. Euclidian distance was the used metric. The optimal number of clusters was estimated with silhouette scores based on the *k-means* algorithm for cluster analysis but was also visually assessed with the dendrogram representation of the fusion sequence. Principal coordinates analysis was subsequently used to visualize individual data in a 2-dimensional scatterplot.

Categorical variables were expressed as counts with percentages, and continuous variables as means with their standard deviation (SD). Categorical variables were compared using the Chi² test. ANOVA tests were used to evaluate the difference between multiple means. All analyses were two-tailed and p-values <0.05 were considered statistically significant for comparative studies.

Predictive modelling was performed using the Orange^©^ data mining software (version 3.35.0) developed by the University of Ljubljana. The JASP^©^ software (version 0.17.2.1) supported by the University of Amsterdam was used for descriptive and frequentist inference statistical analysis.

### Data protection and ethics

Data were anonymized on collection and stored in our institutional electronic repository under the registration number 2023-512 as required by, and in compliance with, French Data Protection Authority (*Commission Nationale de l’Informatique et des Libertés)* guidelines. In accordance with French law, due to its retrospective nature, this study did not require the validation of an Ethics Committee.

## Results

### Characteristics of the study population

**Figure 1:**
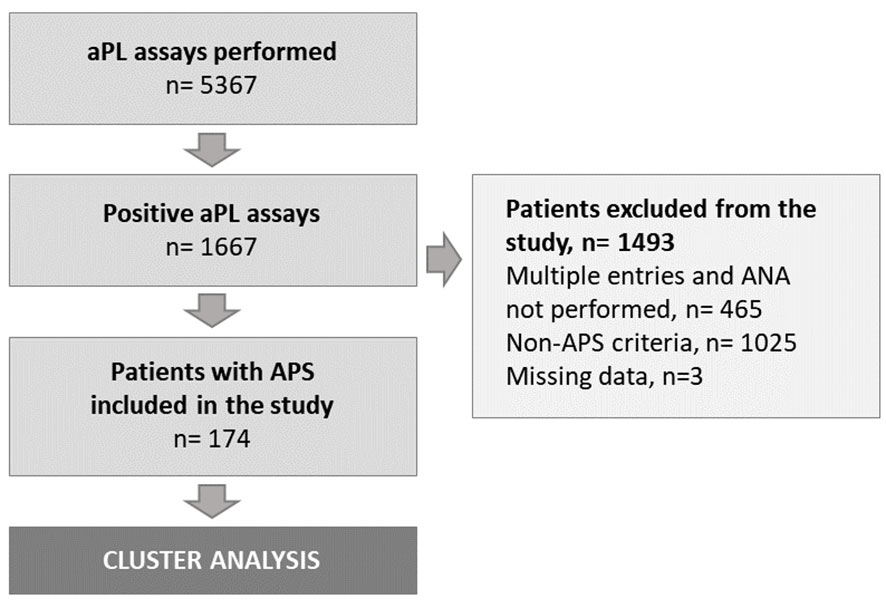
Study flow-chart. aPL, antiphospholipid; ANA, antinuclear antibodies; APS, antiphospholipid syndrome; n, number of subjects.

Over the 90-month study period, 174 patients – that were not only diagnosed with APS but for whom ANA were also performed – were included in the hierarchical analysis (**Figure 1**). Demographics, clinical and biological features at diagnosis are provided in **Table 1**.

**Table 1:** Clinical and biological characteristics of the study population.

	N=174
**Demographics** Age, mean, years ± SD Female-to-male ratio, n/n	50.4 ± 15.4 121/52
**Associated diseases and risk factors** Systemic lupus erythematosus, n (%) Arterial hypertension, n (%) Dyslipidemia, n (%) Diabetes mellitus, n (%) Obesity, n (%) Smoking, n (%)	25 (14) 50 (29) 30 (17) 12 (7) 23 (13) 71 (41)
**Thrombotic events** Arterial (macrovascular), n (%) Venous (macrovascular), n (%) Obstetrical features, n (%)^§^ Cardiac valve involvement, n (%)	86 (49) 57 (33) 34 (20) 7 (4)
Microvascular involvement, n (%) CAPS, n (%) Livedo, n (%) Small-vessel disease of the brain, n (%)	30 (17) 4 (2) 8 (5) 18 (10)
**Antiphospholipid biology** Lupus anticoagulant, n (%) Anticardiolipin IgM antibodies, n (%) Anticardiolipin IgG antibodies, n (%) aβ2-GP.I IgM antibodies, n (%) aβ2-GP.I IgG antibodies, n (%) Double positivity, n (%) Triple positivity, n (%)	133 (76) 56 (32) 114 (66) 25 (14) 136 (78) 36 (21) 44 (25)
**Other laboratory findings** Platelet levels, mean, *10^9^/L ± SD ANA ≥1/160 (positive ANA), n (%) Anti-dsDNA, n (%) Anti-SSA, n (%) C3, mg/dL[N=113] C4, mg/dL [N=113] CH50 activity, U/mL [N=108] Creatinine, mean, µmol/L ± SD eGFR, mL/mn/1.73m², ± SD	246 ± 100 56 (32) 35 (20) 21 (12) 1.37 ± 0.67 0.27 ± 0.19 52.5 ± 17.0 78.6 ± 33.6 91.7 ± 25.7

ANA, antinuclear antibodies; aβ2-GP.I, anti-β2-glycoprotein I; CAPS, catastrophic antiphospholipid syndrome; eGFR, estimated glomerular filtration rate; n, number of subjects; N, overall number for the category; SD, standard deviation; §, percentages calculated exclusively for female subjects.

Antibody titres for aCL and/or aβ2-GP.I (at baseline) were not significantly different between the 4 clusters based on ANOVA testing.

### Hierarchical cluster analysis

**Figure 2:**
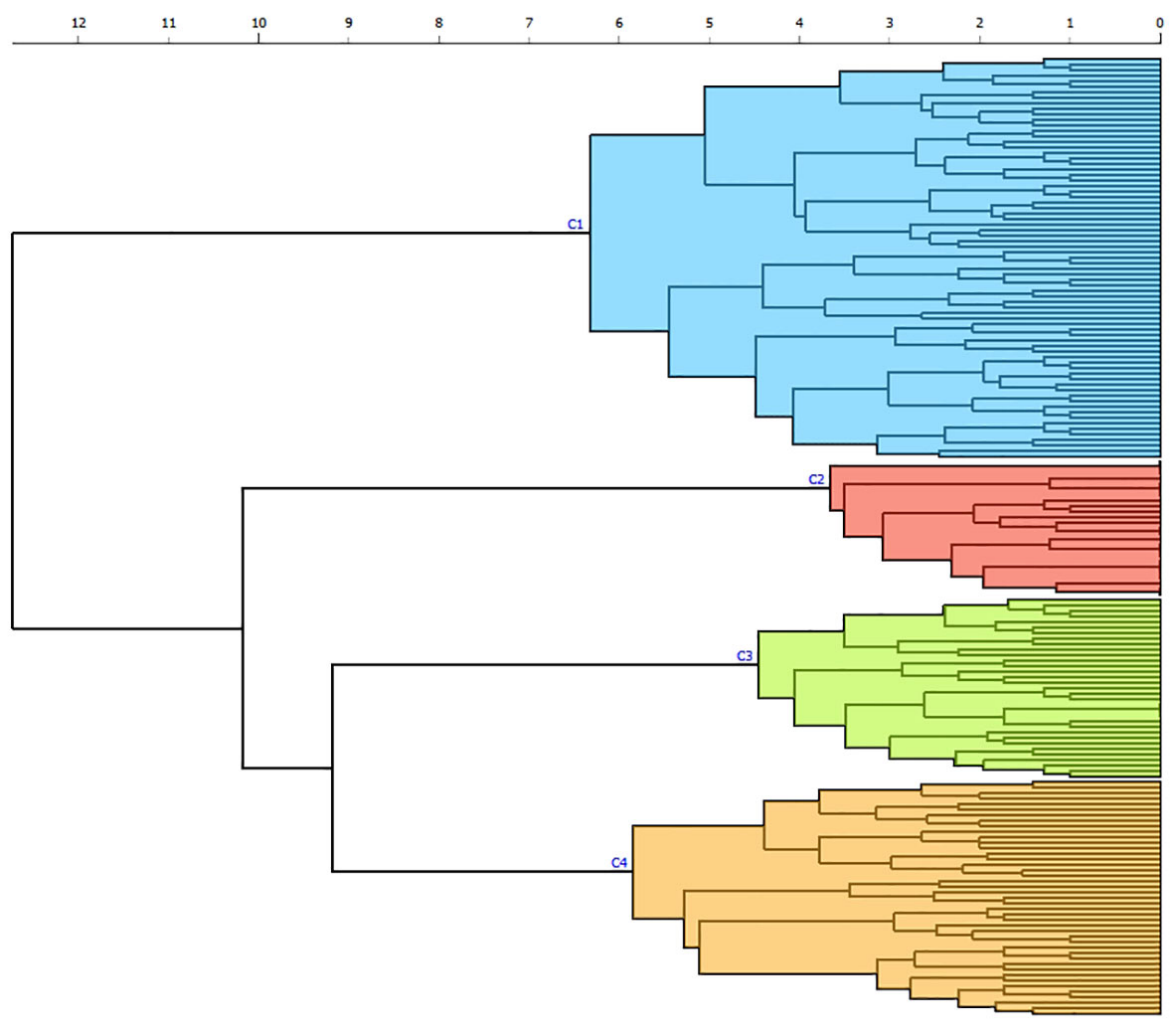
Dendrogram produced through hierarchical clustering of 24 clinical and biological categories in patients with APS. Euclidian distance is reported on the x-axis with horizontal branches reflecting degrees of dissimilarities between combined clusters. Each individual is analyzed on the y-axis, with vertical branches representing the combination of two clusters. Four different clusters (C1 to C4) are individualized by color according to the k-means algorithm: cluster 1 (blue), cluster 2 (red), cluster 3 (green), cluster 4 (orange).

**Figure 3:**
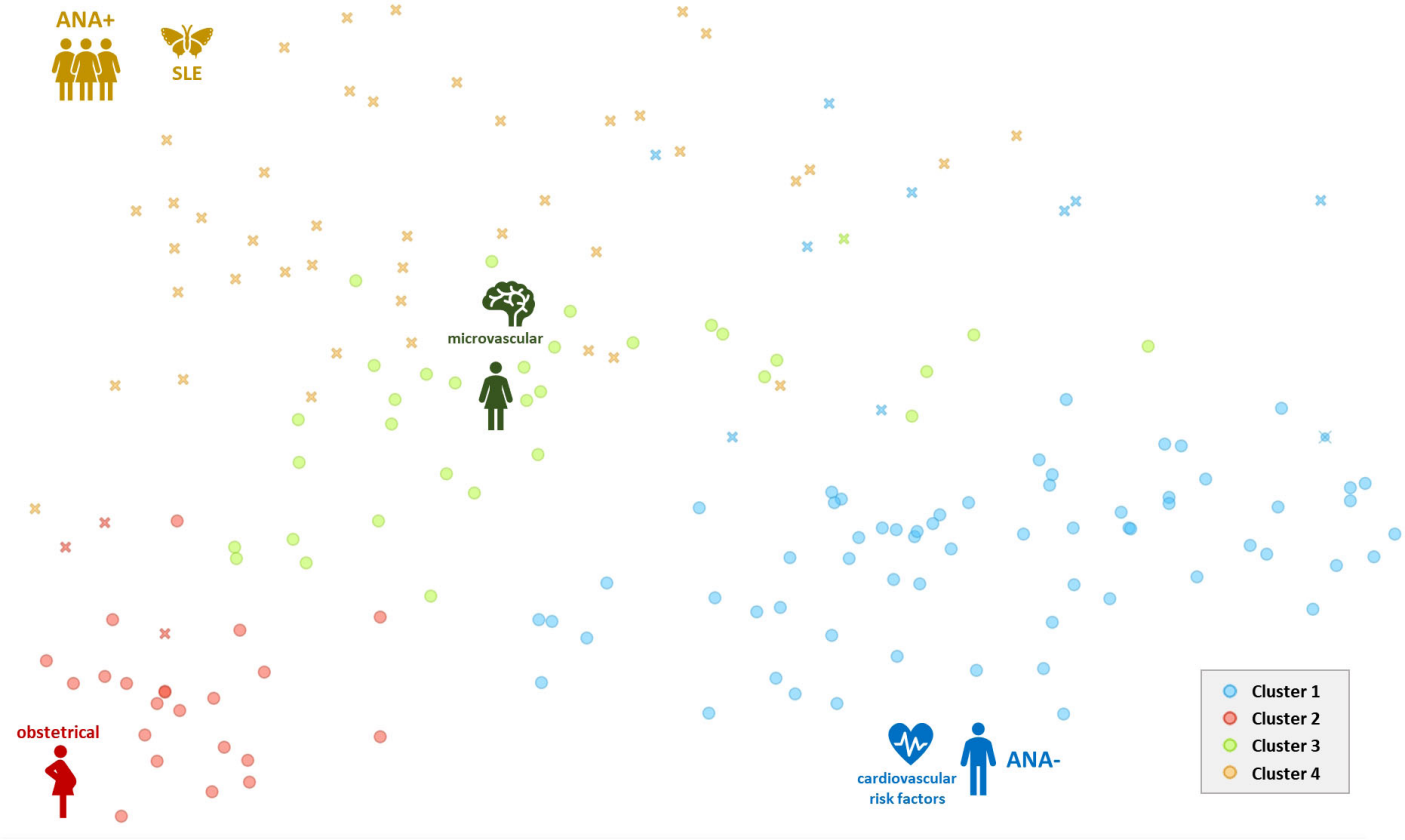
Two-dimensional scatterplot (biplot representation) of results from the principal coordinates analysis with the representation of 4 different clusters of individuals. Crosses (x) represent individuals with positive ANA. Each cluster is represented by a single color. ANA, antinuclear antibodies positivity (+) or negativity (-); SLE, systemic lupus erythematosus.

The *k-means* silhouette score algorithm was able to identify an optimal choice of 4 clusters. These 4 clusters were also visually individualized on the dendrogram (**Figures 2**, **3**). Incomplete data were estimated to be less than 0.1%.

### Cluster description

#### Cluster 1: cardiovascular and arterial risk

This group included 73 patients, with the highest proportion of middle-aged subjects (mean age of 55.8 years) and the lowest proportion of women (n=34). These patients presented with risk factors for cardiovascular disease such as arterial hypertension (48%), hyperlipidemia (36%), obesity (18%) and diabetes mellitus (12%); and 48% were smokers. 69/73 (95%) of patients had arterial thrombotic events at onset. aβ2-GP.I IgG antibodies were present in 86% cases. Arterial thrombotic relapses were observed in one in three patients despite ongoing and long-term anticoagulant therapy (**Table 2**). Most were on vitamin K antagonists (85%).

**Table 2:** Main characteristics of the study population by cluster.

	Cluster 1 (cl.1) n=73	Cluster 2 (cl.2) n=25	Cluster 3 (cl.3) n=33	Cluster 4 (cl.4) n=43	p-value
**Demographics** Age, mean, years ± SD Age>50 years, n (%)^#^ Female gender, n (%)^#^	55.8 ± 14.1 **54 (74)** 34 (47)	39 ± 5.9 0 **25 (100)**	49.8 ± 17.2 18 (54) 26 (79)	48.3 ± 16.1 16 (37) **37 (86)**	<0.01^$^ <0.01 <0.01
**Associated diseases and risk factors** Systemic lupus erythematosus, n (%)^#^ Arterial hypertension, n (%)^#^ Chronic renal disease, n (%) Dyslipidemia, n (%)^#^ Diabetes mellitus, n (%)^#^ Obesity, n (%)^#^ Smoking, n (%)^#^	1 (1) **35 (48)** 3 (4) **26 (36)** **9 (12)** **13 (18)** **42 (48)**	0 0 0 0 0 0 5 (20)	1 (3) 7 (21) 1 (3) 2 (6) 3 (9) 8 (24) 8 (24)	**23 (53)** 8 (15) 2 (5) 2 (5) 0 2 (5) 16 (37)	<0.001 <0.001 - <0.001 0.034 0.010 <0.001
**Thrombotic events** Arterial (macrovascular), n (%)^#^ Venous (macrovascular), n (%)^#^ Obstetrical features, n (%)^§,#^ Cardiac valve involvement, n (%)^#^	**69 (95)** 3 (4) 0 3 (4)	0 0 **25 (100)** 0	2 (6) **32 (97)** 0 2 (6)	**15 (35)** **22 (54)** 8 (22)^§^ 2 (5)	<0.001 <0.001 <0.001 -
Microvascular involvement, n (%)^#^ CAPS, n (%)^#^ Livedo, n (%) Small-vessel disease of the brain, n (%)	10 (14) 0 2 (3) 10 (14)	0 0 0 0	**8 (24)** 0 2 (6) **8 (24)**	**12 (28)** 4 (9) 4 (9) 12 (28)	0.015 0.006 0.250 0.015
**Antiphospholipid biology** Lupus anticoagulant, n (%)^#^ Anticardiolipin IgM antibodies, n (%)^#^ Anticardiolipin IgM titre, MPL ± SE Anticardiolipin IgG antibodies, n (%)^#^ Anticardiolipin IgG titre, GPL ± SE aβ2-GP.I IgM antibodies, n (%)^#^ aβ2-GP.I IgM antibodies, MPL ± SE aβ2-GP.I IgG antibodies, n (%)^#^ aβ2-GP.I IgG antibodies, GPL ± SE Double aPL positivity, n (%) Triple aPL positivity, n (%)	45 (62) 25 (34) 26.6 ± 7.3 48 (66) 100.2 ± 31.3 9 (12) 24.9 ± 11.2 **63 (86)** 100.2 ± 31.3 13 (18) 12 (16)	**20 (80)** 0 2.8 ± 0.4 10 (40) 29.8 ± 11.6 1 (4) 3.0 ± 1.0 8 (32) 29.8 ± 11.6 5 (20) 2 (8)	26 (79) 14 (42) 13.3 ± 3.5 **26 (79)** 111.9 ± 62.7 4 (12) 8.5 ± 3.3 **28 (85)** 111.9 ± 62.7 8 (24) 8 (24)	**42 (98)** 17 (40) 44.0 ± 19.8 **30 (70)** 189.3 ± 67.2 11 (26) 53.0 ± 25.0 **37 (86)** 189.3 ± 67.2 10 (23) **22 (51)**	<0.001 0.002 0.142^$^ 0.018 0.243^$^ 0.073 0.170^$^ <0.001 0.243^$^ 0.849 <0.001
**Other laboratory findings** Platelet levels, mean, *10^9^/L ± SD Thrombopenia <130*10^9^/L, n (%)^#^ Hemolytic anemia, n (%) ANA titre ≥1/160 (positive ANA), n (%)^#^ Anti-dsDNA, n (%)^#^ Anti-SSA, n (%)^#^ Low C3, n (%) [N=113] Low C4, n (%)[N=113] Low CH50, n (%) [N=108] Creatinine, mean, µmol/L ± SD eGFR <60 mL/mn/1.73m², n (%)^#^	246 ± 80 3 (4) 0 9 (12) 7 (9) 1 (1) 2 (3) 3 (4) 10 (14) 85 ± 32 8 (11)	295 ± 140 3 (12) 0 3 (12) 4 (16) 0 0 0 0 68 ± 20 1 (4)	270 ± 75 1 (3) 1 (3) 1 (3) 0 0 2 (6) 3 (9) 5 (15) 70 ± 18 1 (3)	202 ± 101 **9 (21)** 3 (7) **43 (100)** **24 (56)** **20 (47)** 3 (7) 10 (23) 8 (19) 80 ± 47 7 (16)	0.002^$^ 0.011 - <0.001 <0.001 <0.001 - 0.011 0.08 0.055 -
**Treatments** Steroids, n (%) Hydroxychloroquine, n (%) *Antithrombotic agent, n (%)* *Low-dose acetylsalicylic acid, n (%)* *Vitamin K antagonist, n (%)* *DOAC, n (%)* *Immunosuppressants, n (%)*	2 (3) 3 (4) 71 (97) 7 (9) 62 (85) 3 (4) 2 (3)	1 (4) 1 (4) 25 (100) 24 (96) 1 (4) 0 0	1 (3) 3 (9) 31 (94) 0 25 (76) 7 (21) 1 (3)	11 (26) 25 (58) 40 (93) 8 (19) 28 (65) 4 (9) 12 (28)	–
**Outcomes** Arterial relapses, n (%) Venous relapses, n (%)	**24 (33)** 3 (4)	1 (4) 0	4 (12) **13 (39)**	**25 (58)** **13 (30)**	< 0.001 < 0.001

aβ2-GP.I, anti-β2-glycoprotein I; ANA, antinuclear antibodies; aPL, antiphospholipid (biomarker); DOAC, direct oral anticoagulant; eGFR, estimated glomerular filtration rate; n, number of subjects; N, overall number for the category; SD, standard deviation; SE, standard error; ^#^, category used in the hierarchical cluster analysis; ^§^, percentages calculated exclusively for female subjects; ^$^, ANOVA test. When not specified, the p-values refer to Chi-2 testing. Numbers set in boldface highlight the main features of each cluster. Treatments in italics were initiated after the diagnosis of APS.

#### Cluster 2: obstetrical APS

This cluster (n=25) was numerically the smallest and included relatively young female patients (mean age of 39 ± 5.9 years) with obstetrical events, but without a prior history of venous or arterial thrombosis. One in five patients was a smoker but none had associated cardiovascular diseases nor a systemic autoimmune disorder. LA was found in 20 cases and ANA in only three cases. None had hypocomplementemia. Most received low-dose acetylsalicylic acid as long-term therapy with only one relapse (i.e. arterial thrombosis) whilst on this treatment.

#### Cluster 3: venous thromboembolism and microvascular involvement

Cluster 3 (n=33) included predominantly female patients (n=26) with very few cardiovascular risk factors. Most presented with venous thrombosis (97%) at onset. There were no obstetrical features. Small vessel disease of the brain was observed in 8/33 cases but only two were found to have some type of livedo. One in four patients had “double positive” aPL whilst another quarter were “triple positive” for aPL. Relapses were mostly venous (n=13) in a group of patients mostly on vitamin K antagonists (n=25). Of the seven patients on direct oral anticoagulants (DOAC), three relapsed with VTE.

#### Cluster 4: ANA-positive APS

Patients (n=43) in this cluster were mostly female (86%) and were all positive for ANA. More than half presented with SLE (n=23). The highest proportion of “triple positive” patients (51%) was found in this group with LA detected in 42/43 patients. Anti-dsDNA and anti-SSA antibodies were respectively found in 24 and 20 patients. Thrombocytopenia of less than 130*10^9^/L was found in nine patients. More than half was on hydroxychloroquine at the time of APS diagnosis, and a quarter on steroids. Microvascular features were also associated with this group that included all four patients with CAPS.

### Study of relapse rates in relation to ANA positivity

Taken independently, positive ANA were associated with relapse in the overall cohort of APS patients (p<0.01), with a likelihood ratio (LR) of 12.09. Anti-dsDNA and anti-SSA positivity were also found to be associated with relapse with, respectively, LR of 4.166 (p=0.041) and 3.892 (p=0.048). Patients from cluster 4 presented with the highest number of relapses, whether arterial or venous, compared to the three other groups (**Table 2**), regardless of anti-dsDNA and anti-SSA antibody-positivity (p=1 and p=0.740, respectively). Arterial thrombosis was more frequent in ANA-positive patients (**Table 2**).

## Discussion

This study identified four different clusters of patients from a cohort of patients with APS and a complete ANA workup based on an unsupervised clustering method. These phenotypic groups are concisely defined as follows: i) cardiovascular and arterial risk, ii) obstetrical, iii) VTE and microvascular, and iv) ANA-positive APS.

Findings from our study promote the need to include ANA-positivity in the multimodal approach for risk assessment in APS. Most cluster analysis studies on this topic focus on new ways to redefine APS classifications, its diagnosis or to understand disease mechanisms (6–10, 19) (**Supplementary Data**, **Supplementary Table S4**). Few studies, other than ours, have sought to focus on ANA-positivity as a marker of “high risk” APS, outside of SLE (11, 15).

We found that ANA-positivity is associated with a specific phenotype of APS and with higher rates of relapse. To a certain extent, our findings reflect the paradigm shift promoted by the recent 2023 ACR/EULAR APS classification criteria (3). Therefore, we believe that combining the latter with knowledge of ANA status could constitute a novel approach to risk management in APS based on phenotypic patterns.

### Cardiovascular and arterial risk

Patients with cardiovascular disease and aPL positivity are at a high risk of thrombosis as they are likely to have pro-inflammatory endothelial damage in addition to atherosclerosis (5, 20). Such patients also constitute the largest subpopulation amongst subjects with APS as reflected in previous studies (6–11, 19) (**Supplementary Data**, **Supplementary Table S4**). It is therefore hardly surprising that our analysis identified this “cardiovascular risk” group that included mostly middle-aged men with arterial thrombosis. Hypertension and medium/high titres of IgG aCL were also identified as risk factors for initial thrombotic events and echoes previous findings (21). However, one should note that risk-assessment of cardiovascular disease, according to the recent classification criteria, might redefine disease status in previously APS-classified patients (3). This implies that the implementation of anticoagulation in patients with a high-risk profile would depend on future relapses and/or criterion from other clinical domains – if such an approach were to be taken literally. Recurrent thrombosis in APS is difficult to assess with rates ranging from 20% to 30% within the first ten years from disease-onset (5, 22, 23). Studies have suggested that thrombotic patterns do not change during the course of the disease (i.e. relapses are either arterial or venous) and thus highlight the importance of an early characterisation of APS phenotypes (6, 12, 24).

### Obstetrical APS

Our findings also matched those from previous studies relating to obstetrical events (6, 7, 11). Other authors were not able to individualize such a group due to the variables chosen and/or profile of patients included in the analysis (8, 10). This was particularly the case in the study by Nguyen et al. whose study-population was biased by a high proportion of patients with CAPS (10). Based on our findings, one might argue that the “obstetrical” phenotype is associated with a lesser risk of relapse and, in the absence of macrothrombotic events, antiplatelet aggregating agents may suffice. This observation seems to reflect 2019 EULAR guidelines that recommend low-dose acetylsalicylic acid in non-pregnant women with a history of obstetric APS (after risk/benefit evaluation) as well as in pregnant women with a high-risk aPL profile without a prior history of thrombosis nor pregnancy complications (25). Use of low-dose acetylsalicylic acid could also be considered in patients with clinical “non-criteria” obstetric APS (a grade D recommendation) (25). According to EUROAPS data, thrombotic events occur mostly during pregnancy or the puerperium with only a small subset of patients developing SLE over time (i.e. less than 6%) (26).

### VTE and microvascular APS

Our analysis identified a cluster of patients, mostly female, with VTE and venous relapses (cluster 3). Subjects presented with double or triple positive aPL defining a high-risk profile for two thirds of this subpopulation. This group shared similarities with a frequently described phenotype of patients that have triple-positive aPL and VTE (7–10). Caution should be exercised for treating such patients with DOAC in the absence of phenotype-based clinical trials (27, 28).

Cluster 4 was interesting to analyze since it identified ANA-positive patients with high-risk APS. This particular phenotype has been described in previous studies in groups associating either/or: “non-criteria” features, SLE, cytopenias, microthrombotic events and more frequent arterial thrombosis in mostly female patients (7–11) (**Supplementary Data**, **Supplementary Table S4**). ANA-positive APS has been shown to have more “non-criteria” manifestations compared to other forms, an increased frequency of triple-positive aPL and higher rates of relapse (5, 15, 25). In our cohort, thrombosis had the highest rate in this group and occurred despite immunosuppressant drugs, antithrombotic agents and/or hydroxychloroquine intake.

### ANA-positive APS

A prior study found that, of the 43% of patients with SLE with positive aPL, only a third were found to have APS (29). The soon-to-be updated EULAR recommendations regarding the management of SLE-associated APS do not, in these aspects, differ from the 2019 guidelines that state that low-dose acetylsalicylic acid can be prescribed in asymptomatic patients with a high-risk aPL profile (30). In light of findings from previous studies and ours, we believe that ANA-positivity should be acknowledged as a risk factor for potential relapses irrespective of a clinical diagnosis of connective tissue disease. Of note, within this cluster, one out of two patients did not present with SLE. This does not imply that anticoagulation is to be started in asymptomatic aPL-positive patients with ANA, but that clinical and biological work-up should focus on microvascular features and/or cytopenias by, for example, assessing for silent APS nephropathy just as one would for lupus nephritis (31). Therefore close monitoring is warranted, especially considering a propensity for CAPS that is associated with a higher mortality rate primarily due to severe cardiac and cerebral involvement (32). Overlapping and associated pro-thrombotic features (including medication) also need to be addressed (19). Furthermore, patients with ANA-positive APS require optimal anticoagulation with vitamin K antagonists although treatment of isolated VTE with DOAC, in this setting, is still a matter of debate (27, 33).

### Limitations and biases

Our study has limitations – the most important of which, being its retrospective nature that may have introduced a selection bias. The latter may have been increased by the deliberate choice of requiring an immunological work-up with ANA and/or the speciality of the physician ordering the analysis. However, this also appears to be one of its strengths since missing data were extremely limited (i.e. less than 0.1%). Another limit of our study relates to the cluster methodology itself, since clinical and biological changes overtime cannot be assessed. Therefore, one cannot exclude the influence of clinical events and treatments on the “re-categorisation” of patients at a given time. However, based on evidence from long-term registry follow-up, changes in disease course do seem exceptional (12, 24). The quality of the clustering process reflects the very stringent inclusion criteria but it does not exempt us from recognizing an unintentional overlap with unreported prothrombotic factors. Our choice of variables for the cluster analysis was established based on previous studies with an emphasis on clinical categories from the recent APS classification criteria (3, 7–11). Our findings are therefore not only in line with previous initiatives but also refine the categorisation of patients with APS; though they may not be extrapolated to different ethnic groups. Our approach chose to focus on clinically relevant events and therefore “asymptomatic patients” were not considered.

### Clinical significance and perspectives

The clusters that were identified reflect different clinical presentations and risk profiles. From the clinician’s perspective, patient management could be decided according to the predominant phenotype. For instance, obstetrical APS may only require low-dose acetylsalicylic acid as such individuals qualify as part of a “low risk” group (25). However, attitudes would be different if individuals were to present with: “triple positive” aPL, arterial thrombosis, microvascular involvement, cardiac valve thickening/vegetations (28). From our study, one might add ANA-positivity to these criteria. In such “high risk” cases, full-dose anticoagulation with vitamin K antagonists and/or heparin constitute the corner stone of therapy, in keeping with international guidelines (25). In the case of CAPS, triple therapy associating steroids and plasma exchange to anticoagulation, with rituximab as a first-line immunosuppressor with/without follow-up intravenous immunoglobulins whilst preferring cyclophosphamide in cases with positive ANA (34). We would reserve eculizumab for refractory forms of CAPS, especially in documented complement-mediated thrombotic microangiopathy (35). Similarly, future studies studying anticoagulant and immunosuppressive strategies would require adopting a phenotype-based approach.

## Conclusion

This study successfully identified four distinct clusters within APS: i) cardiovascular and arterial risk, ii) obstetrical, iii) VTE and microvascular, and iv) ANA-positive APS. It underscores the importance of microvascular involvement and ANA-positivity in high-risk forms, and categorises a low-risk obstetrical phenotype. It further brings awareness to risk factors for cardiovascular disease that may be confounders for APS in aPL-positive patients but may also constitute a specific phenotype. Finally, our findings promote a novel phenotype-based approach to risk assessment in APS that could hopefully lead to better tailored treatment strategies.

## Data availability statement

The raw data supporting the conclusions of this article will be made available by the authors, on request.

## Ethics statement

Ethical approval was not required for the studies involving humans because this study belongs to the “category 3” of medical research in human participants as defined by the 2016 French law (décret n° 2016-1537 - loi Jardé). The latter states that approval from an ethics committee is not required for retrospective studies (that do not involve any risks). Such is the case of our submission. This information appears in the manuscript. The studies were conducted in accordance with the local legislation and institutional requirements. The participants provided their written informed consent to participate in this study.

## Author contributions

MO: Data curation, Investigation, Writing – original draft, Writing – review & editing. PT: Supervision, Validation, Writing – original draft, Writing – review & editing. BC: Supervision, Validation, Writing – original draft, Writing – review & editing. NM: Conceptualization, Formal analysis, Investigation, Methodology, Software, Supervision, Validation, Writing – original draft, Writing – review & editing.
