# Supplementary material for: Four clinical and biological phenotypes in antiphospholipid syndrome: a cluster analysis of 174 patients with antinuclear antibody tests
Source: Front Immunol. 2024 Feb 19;15:1361062. doi: 10.3389/fimmu.2024.1361062 (PMC10909826; doi:10.3389/fimmu.2024.1361062)
Supplement: Supplementary file 1 [file DataSheet_1.docx]

# SUPPLEMENTARY DATA

### Table S-1. The 2006 Sydney consensus criteria for antiphospholipid syndrome [1].

| **Antiphospholipid antibody syndrome is present**  **if at least one of the clinical criteria and one of the laboratory criteria that follow are met*** | |
| --- | --- |
| **Clinical criteria** | **Laboratory criteria** |
| **1. Vascular thrombosis** One or more clinical episodes of arterial, venous, or small vessel thrombosis, in any tissue or organ.  **2. Pregnancy morbidity**  (a) One or more unexplained deaths of a morphologically normal foetus at/or beyond the 10th week of gestation, with normal foetal morphology documented by ultrasound or by direct examination of the foetus  OR  (b) One or more premature births of a morphologically normal neonate before the 34th week of gestation because of: (i) eclampsia or severe pre-eclampsia defined according to standard definitions, or (ii) recognized features of placental insufficiency  OR  (c) Three or more unexplained consecutive spontaneous abortions before the 10th week of gestation, with maternal anatomic or hormonal abnormalities and paternal and maternal chromosomal causes excluded | **1. Lupus anticoagulant (LA)** present in plasma, on two or more occasions at least 12 weeks apart, detected according to the guidelines of the International Society on Thrombosis and Haemostasis (Scientific Subcommittee on LAs/phospholipid-dependent antibodies)    **2. Anticardiolipin (aCL) antibody of IgG and/or IgM** **isotype** in serum or plasma, present in medium or high titre (i.e. >40 GPL or MPL, or >the 99th percentile), on two or more occasions, at least 12 weeks apart, measured by a standardized ELISA  **3. Anti-β2-glycoprotein-I antibody of IgG and/or IgM isotype** in serum or plasma (in titre >the 99th percentile), present on two or more occasions, at least 12 weeks apart, measured by a standardized ELISA, according to recommended procedures |

**Classification of APS should be avoided if less than 12 weeks or more than 5 years separate the positive aPL test and the clinical manifestation*

### Table S-2. Simplified 2023 ACR/EULAR antiphospholipid syndrome classification (APS) criteria [8]

*(abbreviations) aPL, antiphospholipids; APS, andiphospholipid syndrome; aCL, anti-cardiolipin (antibodies); aβ2-GP.I, anti-β2-glycoprotein I (antibodies); CVD, cardiovascular disease; VTE, venous thromboembolism; w, (gestational) weeks.*

| **ENTRY CRITERIA**  **≥ 1 documented clinical criterion AND ≥ 1 positive aPL test** | |
| --- | --- |
| **CLINICAL DOMAINS AND CRITERIA** | ***weight*** |
| Macrovascular venous thromboembolism (D1)  With a high-risk VTE profile  Without a high-risk VTE profile  Macrovascular arterial thrombosis (D2)  With a high-risk CVD profile  Without a high-risk CVD profile  Microvascular involvement (D3)  Suspected*  Established**  Obstetric (D4)  ≥3 consecutive prefoetal (<10 w) and/or early foetal (<16 w) death  Foetal death (>16 w) in the absence of pre-eclampsia with severe features or placental insufficiency (PI)  Pre-eclampsia (<34 w) or PI with severe features with/without foetal death  Pre-eclampsia (<34 w) and PI with severe features (<34 w) with/without foetal death  Cardiac valve (D5)  Thickening  Vegetation  Haematology (D6)  Thrombocytopenia (lowest 20-130*10^9^/L) | 1  3  2  4  2  5  1  1  3  4  2  4  2 |
| **LABORATORY (aPL) DOMAINS AND CRITERIA** | ***weight*** |
| Lupus anticoagulant positivity (coagulation-based functional assay) (D7)  Single-one time  Persistent (aPL tests at least 12 weeks apart)  aCL and/or aβ2-GP.I positivity^§^ (ELISA) (D8)  IgM only: moderate to high titres  IgG titres that are:  - moderate for aCL and/or aβ2-GP.I  - high for aCL or aβ2-GP.I  - high for aCL and aβ2-GP.I | 1  5  1  4  5  7 |
| **Classify as APS if ≥3 points from clinical domains (D1 to D6) AND ≥3 points from laboratory domains (D7 to D8)**  Only count the highest weighted criterion within each domain  Do not count if there is an equally or more likely explanation than APS | |
| ** livedo racemosa, livedoid vasculopathy (without pathology), aPL nephropathy (no pathology available), pulmonary haemorrhage (symptoms or imaging)*  *** livedoid vasculopathy /with pathology), aPL nephropathy (with pathology), pulmonary haemorrhage (bronchoalveolar lavage or pathology), Myocardial disease (imaging or pathology), Adrenal disease (imaging or pathology)*  *§ moderate (40-79 U) and high (>80 U) levels of aCL/aβ2-GP.I are based on enzyme-linked immunosorbent assays (ELISA)* | |

### Table S-3. Main immunological investigations, with their ranges

|  | **Lower range** | **Upper range** |
| --- | --- | --- |
| Anti-cardiolipin, GPL or MPL | 40 | - |
| Anti-β2-glycoprotein I, GPL or MPL | 40 | - |
| Antinuclear antibodies, titre | 1:80 (negative) | 1:160 |
| Anti-dsDNA (ELISA), IU/mL | 10 | 15 |
| Complement C3 (g/L) | 0,81 | 1,57 |
| Complement C4 (g/L) | 0,13 | 0,39 |
| CH50 complement activity, U/mL | 41.68 | 95.06 |

### Table S-4. Summary of findings from previous cluster analyses on the topic of antiphospholipid syndrome.

*(abbreviations) a**β2-GP.I, anti-β2-glycoprotein I (antibodies); aCL, anticardiolipin (antibodies); anti-ENA, anti-extractable nuclear antigen (antibodies); AIHA, autoimmune haemolytic anaemia; ANA, antinuclear antibodies; aPL, antiphospholipid (antibodies); APS, antiphospholipid syndrome; BMI, body-mass index; CAPS, catastrophic antiphospholipid syndrome; CVA, cerebral vascular attacks (including ischemic strokes); CVD, cardiovascular disease; FPC, foetal pregnancy complications; IUGR, intrauterine growth restriction; LA, lupus anticoagulant; N, number of subjects; PAPS, primary antiphospholipid syndrome; REF., reference; SAPS, secondary antiphospholipid syndrome; SLE, systemic lupus erythematosus; VTE, venous trombo-embolism*

| **REF.** | ***N*** | **Patient selection and chosen variables** | **Clusters** | **Similarities with our study** |
| --- | --- | --- | --- | --- |
| Krause et al.  2007 [10] | 246 | Factor analysis of **only patients with APS**  14 variables: recurrent foetal loss, foetuses with intrauterine growth restriction (IUGR), previous venous thrombotic episodes, previous arterial thrombotic episodes, CVA, epilepsy, migraine, cardiac valvular thickening and/or dysfunction, cardiac valve vegetations, arthritis, livedo reticularis, thrombocytopenia, leukopenia and AIHA. | #1. Cardiac valve abnormalities, livedo reticularis, AIHA, and neurological manifestations  #2. Arthritis, thrombocytopenia and leukopenia  #3. Recurrent foetal loss and IUGR  #4. Arterial thrombosis (few VTE)  #5. Epilepsy and migraine. | #1 is a microvascular phenotype without other autoimmune features found in cluster 4  #2 if combined with #1 is similar to cluster 4  #3 is similar to cluster 2  #4 is similar to cluster 1  #5 is not reflected in our study |
| Zuily et al.  2020 [12] | 497 | Cluster analysis of persistently aPL-positive patients with APS and 10.4% asymptomatic aPL carriers  30 variables: gender, race, arterial thrombosis, VTE, microvascular thrombosis (biopsy), foetal death, FPC, superficial vein thrombosis, transient ischemic attack, livedo reticularis, thrombocytopenia <100*10^9^/L, AIHA, valvular heart disease, nephropathy (biopsy), neuropsychiatric impairment, chorea, seizure, skin ulcer, brain white matter abnormalities, BMI>30 kg/m², hypertension, diabetes mellitus, hyperlipidaemia, smoking, LA, aCL, aβ2-GP.I, SLE, other autoimmune disease | #1. Female patients with no other autoimmune disease, with VTE and triple-aPL positivity (n=179)  #2. Female patients with SLE, VTE, “non-criteria” manifestations, positive LA test, and positive SLE serology (n=180)  #3. Older men with arterial thrombosis, heart valve disease, livedo, skin ulcer, neurological manifestations, and CVD risk factors (n=138) | #1 approximates with cluster 3 (with triple and double-aPL positivity)  #2 is very similar to cluster 4 (including LA positivity)  #3 is very similar to cluster 1 (differs in regard to microvascular involvement) |
| Ogata et al.  2021 [13] | 168 | Cluster analysis of **only patients with APS**  14 variables: age, aPL-score, gender, SLE, hypertension, dyslipidaemia, diabetes mellitus, three or more cardiovascular risk factors, history of arterial thrombosis, history of VTE, positivity for LA, IgG/IgM aCL, IgG/IgM aβ2-GP.I and/or IgG/IgM phosphatidylserine-dependent anti-prothrombin antibodies. | #1. Secondary APS with a majority of SLE patients (72%) (n=61)  #2. CVD risk and arterial thrombosis (n=56)  #3. VTE and triple positivity (n=51) | #1 is similar to cluster 4  #2 is very similar to cluster 1  #3 is similar to cluster 3 (differs in regard to triple-positive aPL) |
| Sciascia et al.  2021 [11] | 486 | Cluster analysis of patients with APS and 27.4% asymptomatic aPL carriers  24 variables: age, female, ethnicity (Caucasian, Black, Hispanic, Asian, Other), PAPS, SAPS, aPL carrier, arterial event, venous event, pregnancy morbidity (defined according to Syndey Criteria), aPL positivity (computed any, double, triple, assessed binary), SLE, SLE ACR criteria manifestation, FPC, severe leukopenia, severe thrombocytopenia (<50*10^9^/L), low C3, low C4, low C3/C4, ANA positivity (≥1:160), anti-dsDNA, anti-ENA, “non-criteria” APS clinical manifestation (as any and per type) | #1. Predominant VTE, triple aPL positivity (n=150)  #2. SLE and ANA positivity, with more frequent arterial thromboses (n=96)  #3. High rate of pregnancy morbidity with obstetric complications (n=79)  #4. Highest rate of cytopenia (n=67)  #5. Asymptomatic aPL carriers (n=94) | #1 has similarities with cluster 3  #2 is similar to cluster 4  #3 is similar to cluster 2  #4 – difficult to assess  #5 – asymptomatic patients were not studied |
| Nguyen et al.  2022 [14] | 509 | Cluster analysis of **only patients with APS**  27 variables: gender, VTE, arterial thrombosis, small vessel thrombosis (biopsy), ≥1 foetal death after 10 weeks, ≥1 premature birth before 34 weeks due to eclampsia, pre-eclampsia, or placental insufficiency, ≥3 consecutive foetal losses before 10 weeks, CAPS, aPL-néphropathy, livedo reticularis, seizures, migraine, chorea, cardiac valvulopathy, SLE, other autoimmune disease, diabetes mellitus, dyslipidaemia, arterial hypertension, AIHA, lymphopenia, thrombocytopenia, LA, aCL, aβ2-GP.I, ANA, low C3 | #1. VTE and premature birth, no autoimmune diseases (n=181)  #2. Older male patients with cardiac valvular disease and CVD risk factors (n=130)  #3. Younger female patients with SLE (or other connective tissue disorders), a history of VTE, cytopenia (n=102)  #4. Microthrombotic features with CAPS, arterial thrombosis, aPL-associated nephropathy (n=96) | #1 is similar to cluster 3 (without premature birth)  #2 is similar to cluster 1  #3 is similar to cluster 4  #4 is not individualised in our analyses |
| Guedon et al.  2023 [15] | 253 | Cluster analysis of persistently aPL-positive patients with APS and 42.3% asymptomatic aPL carriers  35 variables: ANA, aCL and aβ2-GP.I antibodies (IgG et/ou IgM), age, transient ischemic attack, stroke, cancer, CAPS, dyslipidaemia, pulmonary embolism, arterial hypertension, myocardial infarction, Libman-Sachs endocarditis, livedo reticularis, thrombotic microangiopathy, neurologic manifestations, autoimmune cytopenia, aPL nephropathy, ≥3 spontaneous miscarriage before week-10, FPC, obstetric phenotype, aPL carrier, non-criteria manifestations, thrombotic phenotype, female, arterial thrombosis, triple therapy, DVT | #1. Asymptomatic aPL carriers (n=101)  #2. Older male patients with mostly an arterial thrombotic phenotype (n=67)  #3. Young females with an obstetrical phenotype (n=58)  #4. High risk APS, with arterial and venous thrombosis, microvascular features, CAPS (n=27) | #1 – asymptomatic patients were not studied  #2 is similar to cluster 1  #3 is similar to cluster 2  #4 is similar to cluster 4 (triple positive patients, positivity of ANA) |
